# Botox treatment in patients with chronic functional anorectal pain: experiences of a tertiary referral proctology clinic

**DOI:** 10.1007/s10151-019-01945-8

**Published:** 2019-02-16

**Authors:** R. E. Ooijevaar, R. J. F. Felt-Bersma, I. J. Han-Geurts, D. van Reijn, P. F. Vollebregt, C. B. H. Molenaar

**Affiliations:** 1 Department of Gastroenterology and Hepatology, Amsterdam University Medical Centers, Location VU University Medical Centre, PO Box 7057, Amsterdam, 1081 HZ The Netherlands; 2 Department of Anorectal Surgery, Proctos Clinic, Bilthoven, The Netherlands

**Keywords:** Botox, Botulinum toxin type A, BTX-A, Treatment, Chronic anorectal pain, Levator ani syndrome

## Abstract

**Background:**

Anorectal pain is a symptom which may have both structural and functional causes, and can, sometimes, develop into a chronic pain syndrome. Functional causes in particular are challenging to treat when conservative treatment measures fail. Botulinum toxin A (BTX-A) can be applied to relax the anal sphincter and/or levator ani muscle to break the vicious circle of pain and contraction. In our tertiary referral proctology clinic, we evaluated the outcome of patients treated with BTX-A for chronic functional anorectal pain.

**Methods:**

Our electronic database was searched for patients who had BTX-A treatment for chronic functional anorectal pain from 2011 to 2016. All medical data concerning history, treatments, and clinical outcome were retrieved. The clinical outcome (resolution of pain) was scored as good, temporary, or poor.

**Results:**

A total of 113 patients [47 (42%) males; age 51years, SD 13 years, range 18–88 years] with chronic functional anorectal pain were included. The outcome of BTX-A treatment was good in 53 (47%), temporary in 23 (20%), and poor in 37 (33%). To achieve this outcome, 29 (45%) patients needed a single treatment, 11 (44%) a second treatment, and 13 (54%) ≥ 3 treatments.

**Conclusions:**

Chronic functional anorectal pain can be treated successfully with BTX-A in 47% of patients who fail conservative management. Repeated injections may be needed to ensure complete cure in a subgroup of patients.

## Introduction

Anorectal pain is a symptom with a negative impact on the quality of life, and can sometimes develop into a chronic pain syndrome [1]. The etiology can generally be divided into two groups: (1) structural disorders, such as anal fissures, fistulas, or hemorrhoids, disorders often associated with infection, thrombosis, or necrosis which can subsequently lead to pain, and (2) functional disorders, such as levator ani syndrome (LAS) and unspecified anorectal pain [2]. Structural disorders are treated according to their underlying pathology and functional disorders initially with conservative measures such as life style, diet, fibres, laxatives, and pelvic floor physiotherapy. When conservative measures fail, functional disorders can be challenging to treat.

Functional disorders associated with chronic anorectal pain as defined by the Rome IV criteria [3] are LAS, unspecified anorectal pain, and proctalgia fugax [2]. LAS is described as an episode of anorectal pain of unclear etiology, originating in the levator ani muscle. The levator ani muscle is painful during palpation in patients with LAS. The pathophysiology has been related to a hypertonic pelvic floor [2]. Treatments have been unsatisfactory to date, and botulinum toxin A (BTX-A) injections have been applied with limited success [4–6]. Unspecified anorectal pain does not cause any specific tenderness in the levator ani muscle. Proctalgia fugax has a distinct pain pattern and lies beyond the scope of this study. Both structural and functional disorders can lead to pelvic floor dyssynergia (PFD), sometimes called anismus. Patients with PFD typically present with defecation difficulties, such as anorectal pain, prolonged straining, frequent attempts to have a bowel movement and a feeling of incomplete evacuation [5]. The diagnosis of PFD is essentially a clinical diagnosis. Digital rectal examination shows the failure of relaxation or hypertonic state of the external anal sphincter and puborectal muscle during evacuation [7]. According to the Rome IV criteria, additional tests are recommended to confirm the diagnosis; two out of three of balloon expulsion test, defecography, electromyography of the external anal sphincter and puborectal muscle, or anorectal manometry, should be indicative, although the criteria for the latter are not precisely defined [5].

Pelvic floor dyssynergia can be a primary disorder that leads to anorectal pain, but it can also develop secondary to disorders causing anorectal pain. The vicious circle of pain and increased tension of the external anal sphincter and levator ani muscle can lead to even more pain. Patients with PFD are first treated with lifestyle modification, pharmacological, behavioural, and pelvic floor physical therapy including biofeedback. When these treatments are unsuccessful, BTX-A may be offered, although its success has been shown to be limited [4, 5].

BTX-A is the product of the anaerobic bacterium *Clostridium botulinum* [7]. There are seven types of botulinum toxin with different antigenic properties, but all types share a similar structure. BTX-A binds to extracellular glycoprotein structures of the presynaptic cholinergic nerve endings, preventing the secretion of acetylcholine, an excitatory neurotransmitter. The lack of acetylcholine in the synapse causes neuromuscular blockage and muscle paralysis. BTX-A injected into the anal sphincter creates a temporary chemical denervation and injection into the levator, ani induces a similar effect of temporary relaxation The effects of BTX-A last up to 16 weeks [8]. Repeated injections can cause a more rapid breakdown of BTX-A due to the formation of antibodies [8, 9]. BTX-A treats the hypertonia, and, therefore, can help to cure structural disorders such as anal fissure. Subsequently, the normal muscle tone in the external anal sphincter and levator ani muscle can be restored, to break the vicious cycle.

Injection of BTX-A has provided the best results in treating anal fissure, and has been widely accepted with success rates up to 96% [10]. However, the literature shows conflicting results on the treatment of PFD and LAS with BTX-A [4, 11, 12].

The aim of this study was to evaluate the use of BTX-A treatment in patients suffering from chronic functional anorectal pain.

## Materials and methods

### Patients

The electronic database at the Proctos Clinic (tertiary referral proctology clinic, Bilthoven, The Netherlands) was searched for patients who had treatment with BTX-A for chronic anorectal pain between 2011 and 2016. Chronic anorectal pain was defined as pain > 3 months in the anus or pelvic floor according to the Rome IV criteria. All patients with a concomitant structural disorder at the time of BTX-A treatment such as an anal fissure or fistula were excluded. Patients who had had the previous anorectal surgery, including rubber band ligation for hemorrhoids, were also excluded.

At the first visit, a full medical history and physical examination, including a routine digital rectal examination of the anal sphincters and the levator ani muscle, were performed. The combined results of electromyography and digital rectal examination provided a diagnosis of hypertonia of the anal sphincter and/or levator ani muscle. The pain was classified as LAS when the levator ani muscle was painful during palpation. A classification of unspecified anorectal pain was given in the absence of a painful palpation of the levator ani muscle.

Before BTX-A was considered, all patients followed standard conservative treatment, consisting of regulation of bowel movements (dietary advice; prescription of laxatives), pain medication (including opiates and/or pregabalin or amitriptyline if necessary), and psychosomatical counselling if required. All eligible patients had been treated with one or more of these conservative measures for at least 3 months. In addition, patients were seen by a pelvic floor physiotherapist to receive biofeedback. Electromyography of the anal sphincter and levator ani was performed using the MAPle® probe. This probe is able to distinguish muscle tone of individual pelvic floor muscles [13]. Physiotherapy by the pelvic floor physiotherapist was continued throughout treatment.

### Treatment

#### Botulinum toxin injection procedure

Patients with hypertonia of the anal sphincter muscle received 2 injections of 30 units of BTX-A, and patients with hypertonia of the levator ani muscle 2 injections of 40 units each. If patients suffered from hypertonia in both muscles, they received both treatments (2 injections of 30 units into the anal sphincter and 2 injections of 40 units into the levator ani muscle). All injections were given under local anesthesia with the patient in the left lateral position or under general anesthesia with the patient in lithotomy position. The skin around the anus was disinfected. Injections into both the anal sphincter and levator ani muscle were given under digital guidance of one finger which was positioned in the anus. The needle for injection in the anal sphincter muscle was placed laterally to the anus in the inter-sphincteric space and inserted up to 2 cm proximally to inject the BTX-A, depending on the length of the anal canal. The procedure was repeated contralaterally (3 and 9 o’clock). The needle for injection into the levator ani muscle was placed in the skin at 3–4 cm laterally to the anus (lateral to the outer border of the external anal sphincter muscle) and inserted proximally with a slight deviation to the middle of the rectum. The levator ani muscle was hooked up with the tip of the finger in the anus and retracted downwards. The needle was then inserted into the muscle until the tip of the needle could be identified with palpation of the finger inside the rectum near to the rectal mucosa without perforating it. The needle was retracted slightly and BTX-A was injected. The procedure was repeated at the opposite site (4 and 8 o’clock). A total of 4 different surgeons were involved in the care of the patients.

### Follow-up

Patients were reviewed at 3 months after BTX-A treatment. Those with complete resolution of their functional anorectal pain were discharged from further follow-up. Patients with an initial good response to BTX-A treatment but a relapse of their symptoms, or those who had no response or worsening of their symptoms were offered a repeat treatment with BTX-A. Patients with no response to BTX-A were offered up to 3 treatment cycles, and patients with an initial response to treatment up to 8 treatment cycles.

For analysis, patients were further divided into groups based on sex, number of treatments, duration of complaints, hypertonia of the anal sphincter, levator muscle, or both. Informed consent was obtained from all the individual patients in this study. The VU University Medical Centre Ethics Committee approved this study.

### Outcome

The outcome of treatment was scored as good, temporary, or poor. Good was defined as complete resolution of pain and no further treatment or follow-up needed for chronic functional anorectal pain following BTX-A treatment. Temporary was defined as the initial good response but relapse within 3 months. Poor was defined as no improvement, or even worsening of symptoms and anal pain, necessitating treatment with transcutaneous electrical nerve stimulation (TENS), or were referral to a pain specialist. Patients whose outcome was temporary were offered the same options after a relapse of their symptoms and cessation of BTX-A therapy. Up to 8 subsequent treatments with BTX-A were offered.

### Statistical analysis

All statistical analyses were performed using SPSS (IBM, SPSS Statistics 22). Continuous data were described as mean or median depending on the distribution, with standard deviation or range, respectively. To detect differences in outcome between groups, the Chi-square test was performed. If additional testing between subgroups was performed, a Bonferroni correction for the p value was applied. A difference was considered significant if p < 0.05.

## Results

### Patient demographics

A total of 118 of eligible patients were identified, but 5 were lost to follow-up. Thus, 113 patients were evaluated. There were 47 males (42%) and the mean age was 51 years (range 18–88 years). The mean duration of complaints before the first visit to our clinic was 6 years (range 3 months – 40 years). The mean defecation frequency was twice daily. 15 patients (13%) reported obstructed defecation concomitant with their anorectal pain.

Eleven patients (10%) were diagnosed with LAS, while the other 102 (90%) were found to have unspecified anorectal pain. All patients had a form of hypertonia (anal sphincter: 20%, levator ani: 38%, and both muscles: 42%). Eighteen (16%) patients were active smokers. The median number of BTX-A treatments was 2 with a range of 1–8 treatments.

## Outcome

Overall, the outcome was good in 53 (47%) patients, temporary in 23(20%) patients, and poor in 37 (33%) patients. Sex, age, smoking, or duration of complaints did not significantly influence the outcome. Patients with isolated hypertonia of the levator ani (38%) seemed to perform worse when compared to patients with isolated hypertonia of the anal sphincter (20%) or a combination of hypertonia (42%), although these results were not statistically significant (*p* = 0.06). The number of treatments did not influence the overall outcome of treatment (*p* = 0.051), while a relatively large percentage of patients (39%) had a poor outcome following single treatment with BTX-A. The duration of patients’ complaints did not seem to influence the overall outcome (*p* = 0.18), although the proportion of good outcomes among patients with longstanding complaints (≥ 5 years) was substantially lower than among patients with a more recent onset of anorectal pain (28% vs 52% and 48%). There was no difference in overall outcome between patients diagnosed with unspecified anorectal pain or LAS (*p* = 0.72). The results are shown in Table 1. Two patients had temporary fecal incontinence following treatment. No other adverse effects were noted.

**Table 1:** Results of BTX-A treatment in patients with chronic functional anorectal pain

	Number of patients (%)	Good *n* (%)	Temporary *n* (%)	Poor *n* (%)	Chi-square *P* value
All patients	113 (100)	53 (47)	23(20)	37 (33)	–
Sex					
Male	47 (42)	24 (51)	8 (17)	15 (32)	0.69
Female	66 (58)	29 (44)	15 (23)	22 (33)	
Age in years					
18–49	55 (49)	28 (51)	11 (20)	16 (29)	0.67
50 <	58 (51)	25 (43)	12 (21)	21 (36)	
Psychiatric history	12 (11)	8 (66)	2 (17)	2 (17)	0.32*
Smoking	18 (16)	8 (44)	3 (17)	7 (39)	0.81*
Inducing factor known	32 (28)	16 (50)	7 (22)	9 (28)	0.81
Hypertonia					
Sphincter	23 (20)	15 (65)	2 (9)	6 (26)	0.06
Levator ani	43 (38)	13 (32)	12 (32)	18 (36)	
Both	47 (42)	25 (53)	9 (19)	13 (28)	
Number of treatments					
1	64 (57)	29 (45)	10 (16)	25 (39)	0.051
2	25 (22)	11 (44)	10 (40)	4 (16)	
3 or more	24 (21)	13 (54)	3 (13)	8 (33)	
Duration of complaints					
< 1 year	21 (26)	10 (48)	3 (14)	8 (38)	0.18
1–5 years	31 (38)	16 (52)	4 (13)	11 (35)	
5 or more	29 (36)	8 (28)	10 (34)	11 (38)	
Diagnosis					
LAS	11 (10)	5 (46)	3 (27)	3 (27)	0.82
Unspecified anorectal pain	102 (90)	48 (47)	20 (20)	34 (33)	
Obstructed defecation	15 (13)	7 (47)	2 (13)	6 (40)	0.72*

*Compared to patients without stated condition

*BTX-A* botulinum toxin A, *LAS* levator ani syndrome

## Discussion

In this study, treatment with BTX-A provided a sustained cure in 47% of patients suffering from chronic functional anorectal pain. An additional 20% had an initial response to treatment, but relapsed within 3 months. Factors like sex, age smoking, and psychiatric history did not influence outcome. However, these subgroups were small, so no firm conclusions could be drawn. Patients with the isolated hypertonia of the levator ani seem to perform worse than those with solely hypertonia of the anal sphincter or a combination of both, but these results were not statistically significant (*p* = 0.06). The anorectal pain in patients suffering from hypertonia in both muscles might have been caused only by the hypertonic state of the anal sphincter, which could possibly respond better to treatment with BTX-A.

Of 11 patients with LAS, 5 achieved a sustained relief of symptoms following BTX-A treatment. No more than three treatments were given to these patients to achieve this result. We found similar results in a study performed by Bibi et al. [10]. Interestingly, Rao and colleagues found that BTX-A treatment for LAS was not able to relieve pain in any of their patients [11]. We believe that our combined approach of continuous pelvic floor physiotherapy and BTX-A treatment is beneficiary for patients suffering from LAS.

While, in this study, we focused on anorectal pain, many patients in our study might have had PFD, but we did not perform the additional diagnostic tests to confirm PFD according to the Rome IV criteria. Anal manometry is commonly performed to diagnose PFD; however, patterns that are regarded to be abnormal can often be found in healthy volunteers [14]. A digital rectal examination has a high sensitivity as well as positive predictive value as a diagnostic tool in disorders like PFD, if performed by an experienced clinician [15]. Illustrative of the possible effect of BTX-A treatment on PFD are our 15 patients (13%) that reported the symptoms of obstructed defecation as well as anorectal pain. Seven of these patients (47%) achieved sustained resolution not only of their anorectal pain, but also their defecation difficulties. A direct comparison with the study by Emile et al. [4] is not feasible, because we only evaluated the resolution of pain, and did not meet the criteria to diagnose PFD according to the Rome IV criteria. However, resolution of pain might allow these patients to relearn normal defecation behaviour, under the supervision of a pelvic floor physiotherapist.

Patients whose symptoms initially improved but who had a recurrence of their anorectal pain, or showed no improvement, were offered repeated injections after 3 months. Repeated treatment seems to be effective in some patients, which suggests that, sometimes, injections need to be repeated to achieve a better outcome. Patients with longstanding anorectal pain had remarkably less sustained response (28%), although this was not statistically significant in overall outcome (*p* = 0.18). This may in part be due to the development of behavioural changes and sensitisation [1, 16]. Early application of BTX-A treatment in patients with chronic functional anorectal pain might provide a better chance at a sustained resolution of their pain.

In this study, a standard dose of 60, 80, or 140 units of BTX-A was given to each patient based on hypertonia of the anal sphincter and/or levator ani muscle. A recent meta-analysis suggests that a low dose of BTX-A is just as effective as a higher dose when treating an anal fissure, but is associated with fewer complications and side effects [17]. In contrast, a recently published retrospective cohort study with 158 patients not included in the meta-analysis found a higher dose to be more effective, with no difference in side effects [18]. These results might indicate that dosing could still be further optimized, especially for patients suffering from unspecified chronic anorectal pain or LAS. Moreover, we found that only 2 patients developed temporary fecal incontinence, and no other adverse events were reported, indicating that BTX-A treatment is a relatively safe procedure.

The strengths of this study are the large patient population, and the experienced team in a specialized referral centre for proctology. Furthermore, our results show that continuation of physiotherapy may have led to success, because it gave the patient increased awareness of pelvic floor physiology. Limitations are the retrospective nature of the study and the lack of an explicit patient-related outcome measurement (PROM) or visual analogue scale (VAS). There are very few studies reporting on the treatment of chronic functional anorectal pain and outcome is generally scored as clinical response. We believe more prospective studies evaluating the effect of BTX-A treatment in chronic functional anorectal pain are warranted to investigate whether BTX-A treatment should be implemented in the protocol for the treatment of patients with this condition. Furthermore, a validated patient-related outcome score should be developed to validate and interpret results found in future studies.

## Conclusions

Treatment with BTX-A is a safe and reasonably effective treatment for chronic functional anorectal pain. It can provide a sustained resolution of anorectal pain in 47% of patients who are refractory to conservative measures. Repeated treatments with BTX-A after initial treatment failure can still provide a sustained cure in up to 50% of patients. Patients with longstanding duration of anorectal pain might respond less to BTX-A treatment. Patients with anorectal pain should be offered BTX-A treatment after 3–6 months to prevent the development of chronic pain by behavioural changes, sensitisation, and to minimise the impact on daily activities. Patients need to be made aware that anal pain leads to paradoxical contraction of the levator ani muscle and that this might be the onset of the development of chronic anal pain. Continuing pelvic floor therapy after BTX-A may be of added value.
